# Commentary: Chronic SSRI stimulation of astrocytic 5-HT_2B_ receptors change multiple gene expressions/editings and metabolism of glutamate, glucose and glycogen: a potential paradigm shift

**DOI:** 10.3389/fnbeh.2015.00207

**Published:** 2015-08-05

**Authors:** Sophie M. Banas, Silvina L. Diaz, Stéphane Doly, Arnauld Belmer, Luc Maroteaux

**Affiliations:** INSERM UMR S-839, Institut du Fer à Moulin, Université Pierre et Marie CurieParis, France

**Keywords:** serotonin transporter gene, serotonin, neurotransmitter reuptake, depression, mouse models, receptors, cell surface

Working on the serotonin (5-hydroxytryptamine, 5-HT) 5-HT_2B_ receptor since several years, we have read with high interest the review by Hertz et al. (2015). Previous studies from our group demonstrated that a direct injection in mouse raphe nucleus of the 5-HT_2B_ agonist BW723C86 has the ability to increase extracellular levels of serotonin, which can be blocked by the selective 5-HT_2B_ receptor antagonist RS127445 (Doly et al., 2008, 2009). We also reported that an acute injection of paroxetine 2 mg/kg in mice knocked out for the 5-HT_2B_ receptor gene or in wild type mice injected with RS127445 (0.5 mg/kg) triggers a strong reduction in extracellular accumulation of 5-HT in hippocampus (Diaz et al., 2012). Following these observations, we showed that acute and chronic BW723C86 injection (3 mg/kg) can mimic the fluoxetine (3 mg/kg) and paroxetine (1 mg/kg) behavioral and biochemical antidepressant effects in mice (Diaz and Maroteaux, 2011; Diaz et al., 2012).

Hertz and coworkers used some of our published data on mice to support that fluoxetine and other SSRIs are acting as direct 5-HT_2B_ receptor agonists independently of the serotonin transporter (SERT), based on their work on astrocytes. These authors forgot other important informations provided in Diaz's paper (Diaz et al., 2012), i.e., the absence of antidepressant effects of either fluoxetine or BW723C86 in mice lacking either the serotonin transporter (knockout for SERT) or differentiated serotonin neurons (knockout for Pet1). These data (i) rule out that the antidepressant effects of the 5-HT_2B_ agonist BW723C86 or of fluoxetine could be independent of SERT; (ii) they indicate that serotonin neurons expressing SERT (and 5-HT_2B_ receptors) are necessary for the 5-HT_2B_ receptor effects independently of other cell types; (iii) they rule out the possibility that SSRIs mediate antidepressant effects only by stimulating directly putative astrocytic 5-HT_2B_ receptors, which should be intact in these two mutant mice (SERT KO and Pet1 KO). A previous work (Launay et al., 2006) demonstrated a 5-HT_2B_ receptor-mediated control of SERT activity in primary neurons from raphe nuclei, in which the BW723C86/5-HT_2B_ receptor coupling promotes phosphorylations of SERT that can easily explain our findings in mice by a direct regulation of SERT by 5-HT_2B_ receptors in a cell autonomous manner.

Furthermore, Hertz and coworkers state “The ability of all five currently used SSRIs to stimulate the 5-HT_2B_ receptor equipotentially in cultured astrocytes has been known for several years.” This information originates from one of their previous observation on astrocyte cultures that “One micromolar of paroxetine, fluvoxamine or sertraline increased cPLA_2*a*_ expression during chronic treatment; citalopram had a similar effect at 0.1–0.5 μM” (Zhang et al., 2010). Hertz and coworkers validated these data in one of their former review (Hertz et al., 2012), which reproduced their own earlier data (Kong et al., 2002), showing an half maximum effect (competition of mesulergine by fluoxetine on astrocytes) of 1 μM. However, in this review, they say that this initial calculation was “incorrect” and indicated that the Ki value of fluoxetine was in fact 70 nM on putative 5-HT_2B_ astrocytic receptors (Hertz et al., 2012). These values, which are not supported by other published experimental evidence by these authors, are divergent from all published pharmacological values obtained with identified receptors: Bonhaus et al. (1997) and Knight et al. (2004) found Ki values for cloned human 5-HT_2B_ receptors over 10 μM for fluoxetine, norfluoxetine and paroxetine (Table 1).

**Table 1:** **Affinity constants (pKi) for different binding compounds to Human 5-HT_2C_ and 5-HT_2B_ receptors**.

**Compounds**	**5-HT_2B_ Human**		**5-HT_2C_ Human**	
	**pkI**	**Ki (nM)**	**pKi**	**Ki (nM)**
Fluoxetine^b^	< 5	>10,000	6.95	112
Norfluoxetine^b^	< 5	>10,000	7.04	91
Paroxetine^b^	< 5	>10,000	< 5	>10,000
Fluoxetine^k^	< 5	>10,000	6.40±0.03	400
BW723C86^k^	7.33±0.03	47	7.11±0.21	78

*Pharmacological determinations were performed on transfected cells expressing the relevant receptor by heterologous competition. Values are from Bonhaus et al. (1997) (^b^) and Knight et al. (2004) (^k^)*.

To sum up, it is clear from our experiments in mice that acute or chronic fluoxetine (and paroxetine) cannot act by a direct 5-HT_2B_ receptor stimulation independently of SERT and serotonergic neurons. Furthermore, our pharmacological determination in mice is in accordance with affinity of SSRIs for human 5-HT_2B_ receptors with Ki values over 5 μM (Diaz et al., 2012) and with no agonist activity (unpublished), while SSRI Ki values for SERT are in nanomolar range. Many SSRIs have published Ki values around 1 μM for muscarinic acetylcholine and histamine receptors or other monoamine transporters. The contribution of these other receptors/transporters in astrocytes could explain the findings of Hertz and coworkers independently of putative direct agonist effects at 5-HT_2B_ receptors. Finally, the published evidence for 5-HT_2B_ receptor expression in microglia (Krabbe et al., 2011; Kolodziejczak et al., 2015) add another level of complexity, as the concept of the tripartite synapse has recently been expanded to the monoaminergic systems to explain antidepressant drug responses (Quesseveur et al., 2013). A full set of research is still needed to understand the putative role for 5-HT in glial cells including astrocytes and/or microglia in respect to the relationship between SSRIs, serotonergic neurons and 5-HT_2B_ receptors. The needs for further research is warranted to determine how this receptor subtype contribute to SSRI antidepressant effects.

## Sources of funding

Funding for this study was provided by the *CNRS*, the *INSERM*, the *UPMC*, and by grants from the *Fondation de France*, the *Fondation pour la Recherche Médicale* “Equipe FRM DEQ2014039529,” the French Ministry of Research (Agence Nationale pour la Recherche ANR-12-BSV1-0015-01 and the Investissements d'Avenir programme ANR-11-IDEX-0004-02). ML's team is part of the École des Neurosciences de Paris Ile-de-France network and of the Bio-Psy Labex. SD has been supported by fellowships from IBRO and then from *Region Ile de France* DIM STEM.

### Conflict of interest statement

The authors declare that the research was conducted in the absence of any commercial or financial relationships that could be construed as a potential conflict of interest.
